# Non-Invasive Brain Stimulation in Children With Unilateral Cerebral Palsy: A Protocol and Risk Mitigation Guide

**DOI:** 10.3389/fped.2018.00056

**Published:** 2018-03-16

**Authors:** Bernadette T. Gillick, Andrew M. Gordon, Tim Feyma, Linda E. Krach, Jason Carmel, Tonya L. Rich, Yannick Bleyenheuft, Kathleen Friel

**Affiliations:** ^1^Physical Therapy Division, Department of Rehabilitation Medicine, University of Minnesota, Minneapolis, MN, United States; ^2^Department of Biobehavioral Sciences, Teachers College, Columbia University, New York, NY, United States; ^3^Gillette Children’s Specialty Healthcare, Pediatric Neurology, St. Paul, MN, United States; ^4^Courage Kenny Rehabilitation Institute, Minneapolis, MN, United States; ^5^Weill-Cornell Medical College, Blythedale Children’s Hospital, Burke Medical Research Institute, White Plains, NY, United States; ^6^Rehabilitation Science, University of Minnesota, Minneapolis, MN, United States; ^7^Institute of Neuroscience (IoNS), Universite catholique de Louvain, Brussels, Belgium

**Keywords:** cerebral palsy, non-invasive brain stimulation, transcranial magnetic stimulation, transcranial direct current stimulation, repetitive transcranial magnetic stimulation, safety, risk, children

## Abstract

Non-invasive brain stimulation has been increasingly investigated, mainly in adults, with the aims of influencing motor recovery after stroke. However, a consensus on safety and optimal study design has not been established in pediatrics. The low incidence of reported major adverse events in adults with and without clinical conditions has expedited the exploration of NIBS in children with paralleled purposes to influence motor skill development after neurological injury. Considering developmental variability in children, with or without a neurologic diagnosis, adult dosing and protocols may not be appropriate. The purpose of this paper is to present recommendations and tools for the prevention and mitigation of adverse events (AEs) during NIBS in children with unilateral cerebral palsy (UCP). Our recommendations provide a framework for pediatric NIBS study design. The key components of this report on NIBS AEs are (a) a summary of related literature to provide the background evidence and (b) tools for anticipating and managing AEs from four international pediatric laboratories. These recommendations provide a preliminary guide for the assessment of safety and risk mitigation of NIBS in children with UCP. Consistent reporting of safety, feasibility, and tolerability will refine NIBS practice guidelines contributing to future clinical translations of NIBS.

## Background

Neuromodulatory interventions using non-invasive brain stimulation (NIBS) have been increasingly investigated aiming to influence cortical excitability non-surgically in myriad pediatric neurologic conditions including stroke, epilepsy, and unilateral cerebral palsy (UCP) (1). Two forms of NIBS include: (1) electromagnetic induction using transcranial magnetic stimulation (TMS) as an assessment or test of cortical excitability or, when applied repetitively, repetitive transcranial magnetic stimulation (rTMS) as an intervention and (2) electrical current using transcranial direct current stimulation (tDCS) as an intervention. In typically developing children, NIBS has also been investigated as a means to enhance neural plasticity and improve learning (2, 3). As an adjuvant technology, NIBS holds potential to enhance existing pediatric rehabilitation interventions more rapidly and cost-effectively than current practice. However, a consensus surrounding safety, applications, and ethics in the incorporation of NIBS in these conditions has not been established, and guidelines for optimal study design construct are limited. Across four international laboratories, incorporating studies of over 225 children and hundreds of NIBS sessions, we share and integrate current and related pediatric NIBS literature with our protocols in UCP as a means to consider individual variation to mitigate risk and manage adverse events (AEs) in this specific population.

Transcranial magnetic stimulation and rTMS utilize the principles of electromagnetic induction through the use of a coil on the participant’s head over the underlying region of interest. For example, stimulating by placing the TMS coil overlying the area of the primary motor cortex may elicit a motor-evoked potential response in a corresponding hand muscle. If the intent is to test or assess cortical excitability, TMS can be employed in protocols incorporating single or paired pulses (4). This allows for non-surgical assessment of current neurophysiologic status and can be integrated into assessments surrounding neuromodulatory and behavioral interventions. If the intent is to neuromodulate through an intervention, rTMS provides repeated TMS pulses with the aim of inducing a change in cortical excitability: facilitation or inhibition. rTMS, from a neurophysiologic perspective, therefore acts as an intervention to induce action potentials to influence cortical excitability.

Transcranial direct current stimulation applies weak electrical pulses through surface scalp electrodes, at least one anode and cathode, with different montages and dosages (5). Although not strong enough to produce a motor-evoked potential, the current can alter the resting membrane potential, influencing the excitability in endogenous firing rate (6). tDCS may therefore bias cortical activation toward depolarization or hyperpolarization and act as a catalyst to prime the brain for activation and rehabilitation.

Within the context of the developing brain with neurologic injury and underlying neurophysiology, NIBS has been found to influence cortical and behavioral responses. In the case of a unilateral brain injury (e.g., perinatal stroke with resultant UCP) wherein an imbalance between the developing hemispheres may occur, such neuromodulatory interventions may provide a stimulus for integration of dormant yet viable penumbral-region neuronal activity. Added contributions from these ipsilesional neurons may, in turn, allow further development of motor function. In UCP, the application can then either facilitate excitability of one hemisphere (e.g., the lesioned hemisphere) or reduce exaggerated inhibitory effects from an area such as the non-lesioned hemisphere.

In other disorders of cortical excitability, e.g., epilepsy, the target may indeed be the region of the seizure with a stimulation protocol intended to inhibit seizure activity.

Dosing for the forms of interventional NIBS aforementioned varies based on the aims of the protocol and the intent to inhibit or excite by neuromodulation. Dosing has been described in detail in the pediatric population (7). In brief, investigating rTMS in pediatric populations, intensity ranges have been reported to vary between sham settings (0 Hz) and 6 Hz. For tDCS, intensity ranges from sham (0.0 mA) to 2.0 mA settings. For both rTMS and tDCS, the duration of a single session has been reported up to 20 min, with a maximum of 10 daily serial sessions.

Recent computational modeling, investigating dosing and the cortical field induced by NIBS, has guided research protocols with the goal of assessing the safety, feasibility, and efficacy of such interventions in adults and children (8, 9). NIBS can be paired with adjuvant interventions such as upper- and lower-extremity intensive therapies, developing a synergistic application which may advance improvements in the function of the more affected extremities (10). As NIBS mechanisms differ, allowing targeting for specific applications such as priming the nervous system for optimization of therapy (11) or enhancing motor learning (12).

As a means to further explore individual pediatric participant considerations to optimize study design, we investigated the reporting of NIBS-related serious adverse events (SAEs) and minor adverse events (MAEs) in the current pediatric literature, specifically in children with cerebral palsy (CP) inclusive of all subtypes. Table 1 provides a summary of pediatric neuromodulation studies and the reporting of adverse events.

**Table 1:** Summary of pediatric neuromodulation studies and reported adverse events.

Reference	AE addressed	Sample size	Withdraws
**TMS**			

Brouwer and Ashby (13)	No	4 children (10 total with CP, 22 additional controls)	0
Farmer et al. (14)	No	2 (of 4 children)	0
Carr et al. (15)	No	33 (ages 2–26, not defined)	0
Maegaki et al. (16)	Yes	8 children (12 additional either adults or later-onset lesions)	0
Heinen et al. (17)	No	6 children (4 other controls)	0
Maegaki et al. (18)	Yes	17	0
Nezu et al. (19)	No	9	0
Yasuhara et al. (20)	Yes	9	0
Thickbroom et al. (21)	No	7	0
Staudt et al. (22)	No	12	2
Eyre et al. (23)	No	39	0
Berweck et al. (24)	Yes	7 children (10 total with congenital hemiparesis, 8 additional controls)	0
Kuhnke et al. (25)	Yes	9	0
Redman et al. (26)	Yes	22	2
Staudt et al. (27)	No	1 child (8 total)	0
Vry et al. (28)	Yes	15	1
Pilato et al. (29)	No	1	0
Walther et al. (30)	No	7	0
Wilke et al. (31)	No	14	0
Wittenberg (32)	No	10	0
Holmström et al. (33)	Yes	17	0
Kesar et al. (34)	No	13	0
van der Aa et al. (35)	No	37	0
Yang et al. (36)	Yes	5	1
Flamand and Schenider (37)	No	1	0
Islam et al. (38)	No	13 of 16	3
Mackey et al. (39)	No	12	0
Pihko et al. (40)	No	10 children (of a total of 12 children with CP and 12 TDC)	Not stated
Bleyenheuft et al. (10, 41)	Yes	2	0
Narayana et al. (42)	Yes	2	0
Baranello et al. (43)	No	17	0
Friel et al. (44)	Yes	20	0
Kuo et al. (45)	No	20	0

**rTMS**			

Valle et al. (46)	Yes	17	0
Kirton et al. (47, 48)	Yes	10	0
Gillick et al. (8, 49)	Yes	19	0
Kirton et al. (50)	Yes	45	0
Guo et al. (51)	No	1	0
Gupta et al. (52)	Yes	20	0

**tDCS**			

Young et al. (53)	Yes	11	1
Aree-uea et al. (54)	Yes	46	1
Duarte et al. (55)	Yes	24	0
Ferreira et al. (56)	No	12	0
Grecco et al. (57–59)	Yes	24	0
Grecco et al. (57–59)	Yes	20	0
Grecco et al. (57–59)	Yes	1	0
Young et al. (60)	Yes	14	0
Bhanpuri et al. (61)	Yes	6 (of 9 total)	0
Ekici (62)	Yes	1	1
Gillick et al. (63)	Yes	13	1
Grecco et al. (64)	Yes	20	1
Lazzari et al. (65)	No	20	0
Carvalho Lima et al. (66)	Yes	1	0
Grecco et al. (67)	Yes	6	0

*AE, adverse events; CP, cerebral palsy; N/A, not applicable; rTMS, repetitive transcranial magnetic stimulation; tDCS, transcranial direct current stimulation; TDC, typically developing children; TMS, transcranial magnetic stimulation*.

### Existing Safety Guidelines and Protocols

Although summaries and systematic reviews of NIBS studies in both adult and child populations have been published, a consensus as to safe NIBS application in children with UCP, considering brain reorganization, development, and the potential for resultant seizure, has not yet been established (1, 4, 5, 68–73). While the NIBS protocols we have generated feature diagnosis and lesion-specific study designs for children with UCP, we suggest that these recommendations may be applicable as well to NIBS protocols in children with other clinical presentations of CP, such as tetraparesis or diparesis. The indications for such a guide arise from questions as to how NIBS might be applied differently to individuals with UCP based on their lesions or spared neuronal circuitry, as well as from the issue of dosing based on head size and effective current. Additionally, this guide, created from our combined pediatric research, may help to establish optimal practice guidelines and future comprehensive protocols for studies incorporating participants with congenital UCP and postnatally acquired conditions with a clinical presentation of UCP.

*Serious adverse events* reported as related to NIBS are mainly seizure and syncope. TMS-related seizures have been reported directly during or after intervention in children with depression, however, specific to the small samples in pediatric UCP, there have been no reported seizures with either TMS testing or rTMS interventions (1, 70). From the few reports of adults experiencing seizure during single-pulse TMS session applications, many had a pre-existing brain lesion and a diagnosis of intractable epilepsy (68).

Distinct from seizures *induced* by other forms of NIBS, such as direct electrical cortical stimulation (70), tDCS applications are often employed with the intent to *improve* seizure control in both children and adults (74), and typically no SAEs (seizure or syncope) have been reported with tDCS interventions (5). However, a recent report revealed a seizure after a third consecutive session in a daily tDCS protocol in a 4-year-old child with spastic tetraparesis (62). The child, who had a history of seizures and was in the process of adjusting valproate and tapering of topiramate, had been seizure-free for the previous year and reportedly remained seizure-free after the cessation of the tDCS and involvement in the protocol. The direct correlation between tDCS and the incidence of a seizure in this single-case example is difficult to determine.

Two neurocardiogenic syncopal episodes, during initial TMS testing sessions, have been reported in children with UCP (47). In both cases, a history of fainting or previous syncope was reported after the event.

### SAEs Mitigation Recommendations

Seizure management is complex where consensus on one unified approach for selecting and identifying a plan for rescue medications does not exist. Administration of rescue medications requires medically trained caregivers and/or investigators. The selection of rescue medications is typically guided by a pediatric neurologist based on the duration, type, and history of seizures. Several options exist for emergency rescue medication protocols. One option may be emergency administration of rescue medications (e.g., rectal Diastat with a verified appropriate dose based on the child’s weight). Another option is for an alternate rescue medication, such as intranasal Midazolam, which is less intrusive than rectal Diastat (Diazepam) and potentially indicated for children with seizures of a very short duration. A third option is to utilize emergency medical services, allowing for assessment in an emergency room with a physician on staff. Site-specific selection of the seizure action plan depends on (1) recommendations from medical monitor, medical director, and ongoing treating neurologist, (2) training of investigators, and (3) institutional review board (IRB) requirements.

Both seizure management plans and observation records should be established (Appendices A and B in Supplementary Material). If a seizure does occur during study participation, we recommend added documentation for the family and child to represent the isolated seizure as most likely a study-related event. For example, a letter from the study physician (medical director) explaining the NIBS intervention and clarifying the details of the seizure event, may guide follow-up medical management (Appendix C in Supplementary Material).

To minimize risk of syncope, Kirton et al. specifically recommended the following: (1) screening for previous episodes of syncope, fainting history, and evaluation of why the episodes occurred, (2) adequate food/water intake prior to participation in an NIBS study, and (3) monitoring during the study for dizziness and nausea (47).

*Minor adverse events* commonly reported in adults and children during NIBS include headache, dizziness, neck pain, and scalp irritation (1).

### Testing: TMS MAEs

To examine MAE in studies involving children, a literature search yielded 33 studies incorporating TMS in 401 children with CP (13–21, 23–25, 27–36, 38–40, 42–45, 75). Twelve of those studies (36%) addressed or mentioned AEs (Table 1). Four studies (12%) reported a total of nine MAEs, which included discomfort, headache, and decreased tolerance-related directly to the TMS testing (Table 1).

### Intervention: rTMS MAEs

Six rTMS studies have been published using rTMS as an intervention in 112 total children with CP (46, 48, 49, 50–52). Of those six studies, five addressed or mentioned AEs (83%) (Table 1). Four studies (67%) formally reported a total of 57 complaints of MAEs, which included worsening of sleep, social function, mobility, vegetative symptoms, headache, anxiety, dizziness, tingling, mood changes, concentration changes, abnormal muscle contractions, nausea, stomachache, fatigue, and tingling (Table 1). There is wide variability in the reporting of AEs in both real and control groups. It is important to note the percentages of children in the control groups and including assessment of their tolerance of the intervention is central to enrollment and retention of pediatric participants.

### Intervention: tDCS MAEs

Specific to tDCS in children with CP, MAEs commonly include headache, dizziness, and scalp-related complaints (e.g., burning, abrasion, tingling, and itching) (1). Fifteen studies, of a total of 219 children with CP, incorporated tDCS (63, 53–62, 64–67). Thirteen (87%) addressed or mentioned AEs (Table 1). Eleven (73%) reported a total of 48 MAEs, which included complaints of discomfort, rash, burn, itching, sleepiness, tingling, redness, and pain (Table 1).

### MAEs Mitigation

Risk mitigation plans are essential for use by the investigators for preparation and response if an AE, on any level, does occur (Appendices D–F in Supplementary Material). Proper screening and assessment of the potential for MAEs are imperative as these events give clues as to the tolerance a child may have for NIBS. Mitigation begins at enrollment; specific pediatric-based considerations regarding the developmental and clinical status of the child involved in an NIBS protocol include the size and location of the lesion or region of interest, the natural history of the disorder, selection of assessment tools [such as neurologist assessment, e.g., modified Pediatric Stroke Outcome Measure (76)], and an outcome tool [motor function/activity measures, e.g., Assisting Hand Assessment (77)]. Age appropriate questionnaires assessing symptoms can be completed in 5–10 min with children and caregivers. Additionally, we recommend offering breaks/snacks/non-caffeinated beverages, and assessment of nonverbal cues of discomfort (e.g., wincing). Incorporating routine stops during NIBS allows further assessment to further establish if the child feels nauseated, dizzy, or uncomfortable. Careful assessment of AEs, both serious and minor, allows for appropriate management and also for evaluation in the design of potential future interventions.

### AEs Reporting Recommendations

The potential for AEs should be conveyed not only in writing, both on the consent (caregiver) and assent (child) forms, but also verbally. Age-specific and cognitive level appropriate language should be used in AE discussion and interpretation of the scientific literature surrounding NIBS interventions. Integrating feedback from well-informed caregivers before, during, and after the study can be essential to ensure accuracy and completeness of reporting. Inclusion of a quality-of-life measure, depression inventory, or a neuropsychological assessment tool, e.g., Weinberg Depression Scale (78), may be indicated to establish a baseline understanding of the child’s status and potential changes when working with NIBS.

A lack of reporting AEs related to NIBS studies is not necessarily a statement that they did not occur, rather that they may not have been addressed or reported. The comprehensive reporting of such details surrounding an intervention allows not only for potential replication or building upon study findings but also for the opportunity to safely and reliably implement interventions with the intent to benefit patient outcomes (79).

## Study Design Considerations

An understanding of NIBS safety and limitations guides the researcher when constructing optimal pediatric study designs and developing protocols for such analyses. However, studies-to-date vary, without consensus, in establishing participation criteria, monitoring methods, and providing optimal environments. As research progresses toward consensus, we recommend consideration of, at minimum, the following components: criteria for participant safety, safety review committees, control groups, environmental acclimation, and follow-up assessments.

### Study Criteria for Participant Safety

We propose practical consideration, from the outset, of the general study criteria when developing the study design. First, an understanding of the safety contraindications for NIBS is imperative for appropriate participant selection. Contraindications start with identifying indwelling metal and/or medical devices. Case by case review of implanted ferromagnetic and/or medical devices is indicated. We therefore currently devise our safety protocols based upon adult NIBS outcomes and upon an understanding of the effect of NIBS on the underlying cortex. We propose a thorough screening of the child’s medical history, with specific emphasis on diagnosis, history of seizure, syncope, headache, as well as cognitive deficits and behavioral disorders, prior to inclusion in any NIBS study. We have found variability in working with our respective IRBs in terms of study criteria boundaries, but fundamental safety concerns apply when considering candidates for NIBS, whether TMS, rTMS, or tDCS. Table 2 displays the study criteria similarities among our IRB-approved laboratory protocols (Table 2 study criteria).

**Table 2:** Study criteria for NIBS study eligibility for children with CP.

Inclusion criteria	Exclusion criteria
Congenital unilateral cerebral palsy secondary to periventricular leukomalacia or perinatal stroke confirmed by most recent MRI or CT radiologic report with resultant congenital hemiparesis Receptive language function to follow two-step commands A determination by the investigators and institutions, as to the time since last seizure or age since seizure-free, with documentation of all current medications to include anti-epileptic medications Presence of a motor-evoked potential from at least the contralesional hemisphere (if not both hemispheres) for measurement of baseline cortical excitability and as a guide for site of stimulation Able to give informed assent along with the informed consent of a legal guardian Children who have surgeries, which may influence motor function (e.g., tendon transfers). Surgical history should be documented	Metabolic disorders Neoplasm Acquired traumatic brain injury Pregnancy Indwelling metal or incompatible medical devices Evidence of skin disease or skin abnormalities Spasticity injections such as Botulinum Toxin or Phenol Block within the previous 6 months so as not to potentially influence outcomes related to NIBS intervention

*MRI, magnetic resonance imaging, CT, computerized tomography*.

Many of the current guidelines for NIBS in children are derived from the adult stroke literature. However, complete adoption of adult protocols may be inappropriate (e.g., inclusion/exclusion criterion). For example, specific to epilepsy screening and variation between ages, the incidence of epilepsy in adults with stroke is 2.5% (80) whereas in children with UCP, the incidence of epilepsy has been reported at 26% (81). Additionally, based on available data, a child’s risk of seizure recurrence with focal epilepsy has largely stabilized at 2 years of age (82).

Inclusion guidelines for pediatric NIBS participants with a history of epilepsy are not well established in the literature nor does a uniform criterion between pediatric studies exist. The strength of the limited, yet available evidence in establishing guidelines is variable. However, the safety profile for pediatric TMS has recently been reported to be a risk of seizure 0.14% per session (4). Policies established at individual institutions and regulatory oversight mechanisms may reflect different protocols. Indeed, between our own laboratories at different sites, we have discovered variations in the existing study criteria (49, 63, 83). One approach is to include children of any age who have been seizure-free for 2 years. This is based on clinical discussion of medication withdrawal, as well as mechanism of injury, and ongoing EEG positivity (82, 84). Based on the risk-recurrence data available for the child with epilepsy, another approach is to exclude children with any history of seizures *after* 2 years of age (41, 44, 85). This approach includes children who may have only presented with a seizure at the time of brain insult, but who have had no further seizures beyond age 2 years. The approach is also based on the fact that seizure incidence is highest in the first year of life and many children will only have one seizure which occurs at the time of birth (86).

### Safety Review Committee

A designated medical monitor who is not an investigator on the study but who has experience in review of symptoms (e.g., neurologist, pediatric rehabilitation medicine physician) may be included. This monitor could review child status before the study, at a defined interim and after the study is completed. Additionally, the medical monitor can be contacted to review case by case when an AE occurs. A Data Safety Monitoring Board (DSMB) could also be employed with a group of non-study-related professionals whose members (i.e., physicians, statisticians, academicians, and pediatric researchers) are familiar with NIBS and potential AEs, the study design, or congenital UCP. This board can provide interim feedback as to study continuation or cessation and review participant status. Presentation to the DSMB can occur in a blinded manner, with all investigators present, and in an additional unblinded session wherein the unblinded investigators can discuss with the board potential differences between individuals and groups.

### Control Group

The inclusion of a control group of children with and without UCP can allow for group-based comparisons. IRB approval for including children with typical development may depend on the potential to demonstrate direct benefit. Developing an institutional summary of the evidence of NIBS trials in children with typical development displaying the low rate of AEs may aid in gaining IRB approval. Within-participant comparison could occur with crossover designs but may be prohibitive from a feasibility and time-commitment standpoint for families. It has been found, in both adults and children, that participants are unaware of which form of stimulation was received (49, 87). Additionally, if a child with a unilateral brain lesion (e.g., perinatal stroke) is involved in an NIBS intervention targeting one hemisphere or both, the non-lesioned hemisphere and less-affected extremities should be monitored either as a control or a comparison for potential changes in function and safety.

### Acclimation to the NIBS Environment

Through focus polls and feedback from families and children with UCP, our labs have learned that interactions, from the moment the family becomes aware of the study/research, have a strong influence on participation or non-participation decisions. Children and their caregivers need the opportunity to understand the study, directions, and the demands of the protocol. To allow families to acclimate to study-related information, eligibility surveys and follow-up discussions may be conducted over the phone. Allocation of adequate time for consent and assent procedures builds in time for questions and establishes an environment that allows for additional discussion and questions. Children and families considering participation can be connected with past participants of an NIBS study, pending agreement from both families. Additionally, the study environment can be adapted, by pediatric appropriate seating and decoration, to be esthetically pleasing and less anxiety-producing to the child, whenever this opportunity is available.

Pediatric size earplugs provide protection for hearing from the frequency of the TMS (68). Hearing loss was reported in one participant when using an H-coil (88). Due to paucity of research investigating the impact of TMS on pediatric auditory function, we continue to employ and recommend appropriate hearing protection based on the recommendations from Rossi et al. (68), given the developing auditory system as well as the smaller size of the pediatric head resulting in the coil being closer to the ear.

In order for a child to acclimate to tDCS, ramp-up and ramp-down features, as well as a pre-stimulation pulse, can be used. If stimulation thresholds or excitability data are being used as an outcome, ensure that there are no systematic differences in pretest and posttest environments.

### Follow-Up Assessment

Although a child may not have reported or experienced AEs throughout the study, additional feedback from the child/caregiver can improve future study design. A satisfaction survey, such as that adapted from Garvey et al. (89, 90), can assist in analysis of participant satisfaction in the study. A confidential family feedback form, filled out anonymously by the child and caregiver together, can provide helpful information (Appendix G in Supplementary Material). Additionally, formal follow-up at established time points after the study allows long-term assessment of participant status and interpretation of the potential longitudinal effects that NIBS may have (91).

## Conclusion

As evident from the above discussion and AEs, the study design and protocol are crucial elements in guiding NIBS assessments and interventions. Adaptation of supporting equipment and environment may improve the comfort of the child. A thorough medical history, as well as assessment of the caregiver’s and child’s understanding of the protocol, can guide discussions and interpretation of the long-term impact of these interventions. Finally, allowing ample time and opportunity for a child to experience NIBS and give feedback is integral to successful participant enrollment and retention.

NIBS and applications in both typically developing children and those with neurologic diagnoses provide a unique means to establish a “window into the brain,” assessing cortical excitability, mapping, and reorganization. Interventions which incorporate energy transfer to the developing brain, with or without neurologic lesion, must exercise extreme caution not only for the individuals involved but also for advancement of the field. Further considerations include recognition of the anatomic and physiologic differences between an adult brain and a child brain underscoring the need for rigorous investigation before implementation. We present here a comprehensive account of what we have learned to date through previous published research and through collaboration in the work of our laboratories and we offer recommendations for uniformity in reporting of research studies. Key elements include a precise understanding of the organization of the developing brain with a congenital lesion, integration of optimal indications for study participation, incorporation of AEs profile and mitigation, as well as multi-dimensional, longitudinal, study design. Combining our experiences has allowed us to improve our own protocols, with the immediate goal of child safety and the overarching goal to establish a consensus that helps to define best NIBS practice and practice guidelines. Examining both the safety and the feasibility of NIBS studies offers the optimal manner in which to investigate the effectiveness of our interventions.

## Ethics Statement

This study was carried out in accordance with the recommendations of Institutional Review Board with written informed consent from all participants. All participants gave written informed consent in accordance with the Declaration of Helsinki. The protocol was approved by the Institutional Review Board.

## Author Contributions

BG, AG, TF, LK, JC, TR, YB, and KF contributed to the design of this work, writing and revisions of this work, final approval, and agreed to be accountable for all aspects of the work.

## Conflict of Interest Statement

The authors declare that the research was conducted in the absence of any commercial or financial relationships that could be construed as a potential conflict of interest. The reviewer RF and handling editor declared their shared affiliation.

## Supplementary Material

Appendix A Seizure Management.

Appendix B Seizure Observation Record.

Appendix C Seizure Event Letter.

Appendix D Transcranial Direct Current Stimulation (tDCS) Risk Mitigation.

Appendix E Transcranial Magnetic Stimulation (TMS) Risk Mitigation.

Appendix F Participant Report of Symptoms.

Appendix G Confidential family feedback form.
