# Supplementary material for: Non-Invasive Brain Stimulation in Children With Unilateral Cerebral Palsy: A Protocol and Risk Mitigation Guide
Source: Front Pediatr. 2018 Mar 16;6:56. doi: 10.3389/fped.2018.00056 (PMC5864860; doi:10.3389/fped.2018.00056)
Supplement: Appendix A — Seizure Management. [file Data_Sheet_1.ZIP › Appendix_A.DOCX]

Supplementary Material

**Non-Invasive Brain Stimulation in Children with Unilateral Cerebral Palsy:**

A Protocol and Risk Mitigation Guide

Gillick BT^1*^, Gordon AM^2^, Feyma T^3^, Krach LE^4^, Carmel J^5^, Rich TL^6^, Bleyenheuft Y^7^, Friel K^5^

*** Correspondence:** Bernadette T. Gillick, Ph.D., MSPT, PT [gillick@umn.edu](mailto:gillick@umn.edu)

**Appendix A: Seizure Management Guidelines**

Safety and proper care of an individual having a seizure must be of utmost concern. It is unlikely that a seizure will occur during the study, yet if one does occur; it is likely to be self-limited. Observation, documentation, and management of all events will be performed by trained study personnel. The study Medical Director will be notified of the testing and intervention schedules, and will be on call for comprehensive seizure management.

Response guidelines are as follows:

**Proper Guidelines for Seizure Care**

1. Recline the chair to lay the person down. Protect the participant from falling.
2. Remove harmful or hard objects from the surrounding area.
3. Loosen restrictive clothing around the neck.
4. Do not restrain movements unless it is necessary to keep the person safe.
5. Do NOT place anything in the person’s mouth.
6. If the person is having breathing difficulties, assist them to lying on their side.
7. Remain calm and reassure the person and observers of safety during and after the seizure.
8. The testing investigator will remain with the participant throughout the event. Prior to the testing and intervention sessions, a member of the on-site investigative team will be assigned the task of calling for emergency assistance if determined, along with contacting the study medical director. The treating investigator should obtain help if indicated. If a medical emergency or further assistance is required, the participant’s study chart will be available with emergency contact information.
9. Observe and record what occurred during the seizure (see below in Guidelines and Appendix E), how long it lasted, and if any injury occurred.
10. Continue to observe the participant throughout the duration of the seizure for safety. If the seizure does not abate within 5 minutes or more than one seizure occurs in succession, emergency intervention will be undertaken.
11. Length of Seizure. If the seizure lasts less than 5 minutes.
    1. After the seizure has ceased, roll the participant into the recovery position, ensure the participant has respiratory and circulatory function, and continue to observe the participant while consciousness is regained.
    2. Assess whether the participant is experiencing any other side effects (dizziness, etc.) and if the participant appears normal and reports feeling normal, arrange for ambulance transportation for the participant to the Emergency Room (ER) for evaluation. The legal guardian and the Principal Investigator must accompany the participant to the ER.
    3. Provide the participant with a letter documenting that a seizure was experimentally induced.
12. Length of Seizure. For prolonged seizures, longer than 5 minutes, the following intervention will be undertaken:
    1. Care as above and participant will be transported to the emergency room for further evaluation. Follow-up electroencephalogram (EEG) and formal neurologic evaluation is indicated.
    2. The principal investigator (PI) will follow up with the participant the following day to ensure no other adverse effects have been experienced.

**Guidelines for Seizure Observation**

The following are recommendations on what to document.

1. Before the seizure - Look for a change in mood hours or days before the seizure. Report any noted warning or aura such as a strange feeling, tingling, nausea, strange taste or smells, ringing in ears, vision changes, headache, or a sudden emotion (fear, anxiety, or pleasant feeling).
2. During the seizure and possible triggers- Change in awareness, confusion, memory, unconscious state. Change in speech- clear speech, responds only with a few words or sounds, speech doesn’t make sense, unable to talk. Change in facial expression- staring, blinking, drooling. Jerking movements or turning of the head, eyes, or body. Changes in muscle tone- stiff or limp. Twitching or jerking of face, arms, legs. Lip-smacking, chewing, swallowing, picking at clothes or rubbing of hands, tapping of feet. Walking around or continuing an action confused or unaware. Falling. Changes in color of skin, sweating, breathing. Loss of urine or bowel control.
3. What part of the body was directly involved in the seizure.
4. The person’s behavior after the seizure. Able to respond to voice or touch. Aware of name, place, time, memory of the seizure, able to talk, weakness or numbness in arm or leg, mood sleepiness.
5. Length of seizure and time before person resumes normal activity.

(Developed by incorporation of material from the Gillick Pediatric Laboratory, the American Academy of Neurology, 2015 (aan.com), Berenson-Allen Center for Non-Invasive Brain Stimulation, and Gillette Children’s Specialty Health Care Guidelines)
