# Supplementary material for: Non-Invasive Brain Stimulation in Children With Unilateral Cerebral Palsy: A Protocol and Risk Mitigation Guide
Source: Front Pediatr. 2018 Mar 16;6:56. doi: 10.3389/fped.2018.00056 (PMC5864860; doi:10.3389/fped.2018.00056)
Supplement: Appendix A — Seizure Management. [file Data_Sheet_1.ZIP › Appendix_B.DOCX]

Supplementary Material

**Non-Invasive Brain Stimulation in Children with Unilateral Cerebral Palsy:**

A Protocol and Risk Mitigation Guide

Gillick BT^1*^, Gordon AM^2^, Feyma T^3^, Krach LE^4^, Carmel J^5^, Rich TL^6^, Bleyenheuft Y^7^, Friel K^5^

*** Correspondence:** Bernadette T. Gillick, Ph.D., MSPT, PT [gillick@umn.edu](mailto:gillick@umn.edu)

**Appendix B- Seizure Observation and Documentation Record**

Participant ID # ____________ Date: ___________ PI: ___________

Investigator signature reporting this event: ______________________________

Printed Investigator Name reporting this event: ______________________________

Seizure Event

What exact time did the seizure event begin? ___________ AM or PM (circle one)

What exact time did the seizure event end? ___________ AM or PM (circle one)

What was the estimated total active seizure duration? __________ seconds or minutes (circle one)

Did any injury occur? Yes or No (circle one)

If yes, please describe:

Observation of seizure related events

| **PRE SEIZURE** | |  | **DURING SEIZURE** | |  | **POST SEIZURE** | |
| --- | --- | --- | --- | --- | --- | --- | --- |
| **Behavior** | **Check if Yes** |  | **Behavior** | **Check if Yes** |  | **Behavior** | **Check if Yes** |
| Warning signs (e.g., aura) |  |  | Lack of awareness |  |  | Response |  |
| Sensation (e.g., tingling) |  |  | Speech changes |  |  | Aware of name |  |
| Nausea |  |  | Facial expression change |  |  | Aware of place |  |
| Strange taste or smells |  |  | Muscle tone change |  |  | Aware of time |  |
| Ringing in ears |  |  | Abnormal movements (body part) |  |  | Memory of the seizure |  |
| Vision changes |  |  | Confusion |  |  | Able to talk |  |
| Headache |  |  | Falling |  |  | Weakness or numbness |  |
| Sudden emotion (e.g., fear, anxiety) |  |  | Skin color/sweating, breathing changes |  |  | Mood changes |  |
| Other (list): |  |  | Bowel/bladder continence |  |  | Bowel/bladder continence |  |
| Other (list):  Please further describe any checked boxes above: |  |  | Please further describe any checked boxes above: | |  | Sleepiness |  |
|  |  |  |  |  |  | Please further describe any checked boxes above: | |
|  | |  |  |  |  |  |  |
