# Supplementary material for: Non-Invasive Brain Stimulation in Children With Unilateral Cerebral Palsy: A Protocol and Risk Mitigation Guide
Source: Front Pediatr. 2018 Mar 16;6:56. doi: 10.3389/fped.2018.00056 (PMC5864860; doi:10.3389/fped.2018.00056)
Supplement: Appendix A — Seizure Management. [file Data_Sheet_1.ZIP › Appendix_C.DOCX]

Supplementary Material

**Non-Invasive Brain Stimulation in Children with Unilateral Cerebral Palsy:**

A Protocol and Risk Mitigation Guide

Gillick BT^1*^, Gordon AM^2^, Feyma T^3^, Krach LE^4^, Carmel J^5^, Rich TL^6^, Bleyenheuft Y^7^, Friel K^5^

*** Correspondence:** Bernadette T. Gillick, Ph.D., MSPT, PT [gillick@umn.edu](mailto:gillick@umn.edu)

**Appendix C- Seizure Event Letter**

Date:

Time:

To Whom It May Concern,

I hereby certify that ___________________________ has experienced a single seizure while participating in an experimental study employing non-invasive brain stimulation, transcranial magnetic stimulation (TMS) and/or transcranial direct current stimulation (tDCS). TMS and tDCS are non-invasive methods of neuromodulation. The seizure experienced by this participant occurred during the __________ session. There are no previous documented events of seizure due to these forms of non-invasive brain stimulation. However, the seizure this participant experienced is most likely a direct consequence of this exposure. From a neurological perspective, this is to be considered an induced, precipitated seizure, yet should be formally evaluated. As part of standard medical care and follow-up for a first seizure, the participant has been evaluated at _____________________________. This participant is not known to be epileptic yet does have congenital unilateral cerebral palsy. There is no medical basis to consider this seizure a risk factor in return to school activity, employability or medical insurability.

We are providing this letter in compliance with the XXX (State University or Organization) Clinical and Translational Science Institute and the Institutional Review Board. Please feel free to contact me if you have any other questions or concerns.

Sincerely,

Signature

____________________________________________________________________________

Printed Name

____________________________________________________________________________

Title

____________________________________________________________________________

Clinic/Organization

Phone
