# Supplementary material for: Non-Invasive Brain Stimulation in Children With Unilateral Cerebral Palsy: A Protocol and Risk Mitigation Guide
Source: Front Pediatr. 2018 Mar 16;6:56. doi: 10.3389/fped.2018.00056 (PMC5864860; doi:10.3389/fped.2018.00056)
Supplement: Appendix A — Seizure Management. [file Data_Sheet_1.ZIP › Appendix_D.DOCX]

Supplementary Material

**Non-Invasive Brain Stimulation in Children with Unilateral Cerebral Palsy:**

A Protocol and Risk Mitigation Guide

Gillick BT^1*^, Gordon AM^2^, Feyma T^3^, Krach LE^4^, Carmel J^5^, Rich TL^6^, Bleyenheuft Y^7^, Friel K^5^

*** Correspondence:** Bernadette T. Gillick, Ph.D., MSPT, PT [gillick@umn.edu](mailto:gillick@umn.edu)

**Appendix D**

**Risks and Mitigation of Risks Transcranial Direct Current Stimulation (tDCS)**

There are no significant risks associated with tDCS or serious adverse events anticipated in this study.(1-3) Due to the indications of the procedure, electrical currents will however be passed into the cortex, and minor adverse effects may occur. Adverse effects associated with transcranial direct current stimulation are minimal and may include itching, tingling, burning sensation in the area of the electrodes, headache, neck pain, scalp pain, skin redness, fatigue, trouble concentrating, and acute mood change. Current intensity, current duration, and electrode size, skin integrity, voltage and tolerance will all be continuously assessed and are paramount in this study in children with congenital unilateral cerebral palsy. Specific anticipated risks and risk mitigation procedures are listed in the table below.

| **Study Procedure** | **Anticipated Risks** | | **Risk Mitigation** |
| --- | --- | --- | --- |
| tDCS | Burn- Electrolysis | | Ensure proper electrode contact with skin |
| tDCS | Stimulation in participants with reduced sensation | | Assess sensation, avoid placing electrodes over areas of decreased sensation |
| tDCS | Stimulation over broken skin, reduced resistance | | Assess skin integrity, avoid placement of electrodes over recent shaving, skin defects |
| tDCS | Stimulation over conductive implants | | Screen appropriately for exclusion criteria of implants |
| tDCS | Stimulation over a tumor which may alter metabolic activity | | Screen appropriately for exclusion criteria of neoplasm. |
| tDCS | Threshold altering pharmacologic agent | | Physician review of each medical record for determination of appropriateness for study inclusion. |
| tDCS | Itching, Tingling, Burning Sensation in the area of the electrodes | | Ensure proper contact of surface electrodes with skin. Maintain current dosage within low-range of researched dosages. Ensure that electrode sponges are properly sanitized and that saline solution is appropriately employed. |
| tDCS | Headache | | Ensure that headband securing electrodes is in proper placement, yet not to the level of impingement of scalp area. Maintain current dosage within low range of delivery. |
| tDCS | Pain-Neck, Scalp | | Ensure that electrodes are in proper contact with  skin and adjust head position as needed for comfort. |
| tDCS | | Skin Redness | Ensure proper electrode position and proper level of moisture to even stimulation across the electrode |
| tDCS | | Fatigue, Sleepiness | Screen for continuous effect at follow-up visit. |
| tDCS | | Concentration or Mood Changes | Evaluate cognitive status through physician examination and psychometric testing at three time points. |

**References**

(1) Bolognini N, Pascual-Leone A, Fregni F. Using non-invasive brain stimulation to augment motor training-induced plasticity. J Neuroeng Rehabil 2009 Mar 17;6:8.

(2) Bolognini N, Vallar G, Casati C, Latif LA, El-Nazer R, Williams J, et al. Neurophysiological and Behavioral Effects of tDCS Combined With Constraint-Induced Movement Therapy in Poststroke Patients. Neurorehabil Neural Repair 2011 NOV-DEC;25(9):819-829.

(3) Brunoni A, Amadera J, Berbel B, Volz M, Rizzerio B, Fregni F. A systematic review on reporting and assessment of adverse effects associated with transcranial direct current stimulation. International journal of neuropsychopharmacology 2011;14(8):1133-1145.
