# Supplementary material for: Non-Invasive Brain Stimulation in Children With Unilateral Cerebral Palsy: A Protocol and Risk Mitigation Guide
Source: Front Pediatr. 2018 Mar 16;6:56. doi: 10.3389/fped.2018.00056 (PMC5864860; doi:10.3389/fped.2018.00056)
Supplement: Appendix A — Seizure Management. [file Data_Sheet_1.ZIP › Appendix_E.DOCX]

Supplementary Material

**Non-Invasive Brain Stimulation in Children with Unilateral Cerebral Palsy:**

A Protocol and Risk Mitigation Guide

Gillick BT^1*^, Gordon AM^2^, Feyma T^3^, Krach LE^4^, Carmel J^5^, Rich TL^6^, Bleyenheuft Y^7^, Friel K^5^

*** Correspondence:** Bernadette T. Gillick, Ph.D., MSPT, PT [gillick@umn.edu](mailto:gillick@umn.edu)

**Appendix E**

**Risks and Mitigation of Risks Transcranial Magnetic Stimulation (TMS)**

There are no serious adverse events anticipated in this study using single-pulse TMS.(1-3) However, due to the indications of the TMS testing session, electrical currents will be passed into the cortex, and minor adverse effects may occur. The most common minor adverse event we have found in our studies has been transient, self-limiting headache. (4) Stimulation intensity, number of pulses and tolerance will all be continuously assessed and are paramount in this study in children with congenital unilateral cerebral palsy. Specific anticipated risks and risk mitigation procedures are listed in the table below.

| **Study Procedure** | **Anticipated Risks** | **Risk Mitigation** |
| --- | --- | --- |
| Stereotactic Neuronavigation Measurement for TMS Coil Placement | Perception of touching skin with tip of neuronavigation pointer. | Guided pointer placement with investigator holding pointer steady during measurement. |
| TMS | Stimulation over a tumor which may alter metabolic activity | Screen appropriately for exclusion criteria of neoplasm. |
| TMS | Threshold altering pharmacologic agent | Physician review of each medical record for determination of appropriateness for study inclusion. |
| TMS | Headache | Screen for continuous effect throughout session through planned and spontaneous inquiry as well as invitation to report discomfort at any time. |
| TMS | Fatigue, Sleepiness | Screen for continuous effect throughout session through planned and spontaneous inquiry as well as invitation to report discomfort at any time. |
| TMS | Temporary mild hearing loss due to noise level of equipment | Ear plugs will be inserted before commencement of TMS application. |

**References**

(1) Rossi S, Hallett M, Rossini PM, Pascual-Leone A, Safety of TMS Consensus Group. Safety, ethical considerations, and application guidelines for the use of transcranial magnetic stimulation in clinical practice and research. Clin Neurophysiol 2009 Dec;120(12):2008-2039.

(2) Quintana H. Transcranial magnetic stimulation in persons younger than the age of 18. J ECT 2005 Jun;21(2):88-95.

(3) Krishnan C, Santos L, Peterson M, Ehinger M. Safety of Noninvasive Brain Stimulation in Children and Adolescents. Brain stimulation 2015;8(1):76-87.

(4) Gillick B, Krach L, Feyma T, Rich T, Moberg K, Menk J, et al. Safety of primed repetitive transcranial magnetic stimulation and modified constraint-induced movement therapy in a randomized controlled trial in pediatric hemiparesis. Arch Phys Med Rehabil 2014.
