# Supplementary material for: Non-Invasive Brain Stimulation in Children With Unilateral Cerebral Palsy: A Protocol and Risk Mitigation Guide
Source: Front Pediatr. 2018 Mar 16;6:56. doi: 10.3389/fped.2018.00056 (PMC5864860; doi:10.3389/fped.2018.00056)
Supplement: Appendix A — Seizure Management. [file Data_Sheet_1.ZIP › Appendix_F.DOCX]

Supplementary Material

**Non-Invasive Brain Stimulation in Children with Unilateral Cerebral Palsy:**

A Protocol and Risk Mitigation Guide

Gillick BT^1*^, Gordon AM^2^, Feyma T^3^, Krach LE^4^, Carmel J^5^, Rich TL^6^, Bleyenheuft Y^7^, Friel K^5^

*** Correspondence:** Bernadette T. Gillick, Ph.D., MSPT, PT [gillick@umn.edu](mailto:gillick@umn.edu)

**Appendix F - Participant Report of Symptoms**

Participant ID: PI:

**Type of Non-Invasive Brain Stimulation: __TMS ___rTMS ___tDCS ___Other (Describe):______________**

| **Visit Date:** | | **PRE** Time: Investigator: | | **Mid-Intervention**  **(if applicable)**  **Time:** | **POST** Time: Investigator: | |
| --- | --- | --- | --- | --- | --- | --- |
| **How are you feeling overall right now?** | Participant |  | | **How is the stimulation feeling right now?** |  | |
|  | Caregiver |  | |  |  | |
| **“Right now, do you feel you have or are……?”** | | **Value**  **(per child)**  1 absent  2 mild  3 moderate 4 severe | **Relation (per investigator)**  1 unrelated  2 unlikely  3 possible 4 probable  5 definite |  | **Value**  **(per child)**  1 absent  2 mild 3 moderate  4 severe | **Relation (per investigator)**  1 unrelated  2 unlikely  3 possible 4 probable  5 definite |
| Headache | |  |  |  |  |  |
| Unusual feelings on the skin of your head | |  |  |  |  |  |
| Neck pain | |  |  |  |  |  |
| Tingling | |  |  |  |  |  |
| Itchiness | |  |  |  |  |  |
| Sleepiness | |  |  |  |  |  |
| Difficulty paying attention | |  |  |  |  |  |
| Unusual /feelings, attitude, emotions | |  |  |  |  |  |
| Tooth pain | |  |  |  |  |  |
| Change in hearing | |  |  |  |  |  |
| Nausea/Sick to Stomach | |  |  |  |  |  |
| Unusual twitches or movements in muscles | |  |  |  |  |  |
| Dizziness | |  |  |  |  |  |
| Anxious/Worried/Nervous | |  |  |  |  |  |
| Forgetful | |  |  |  |  |  |
| Difficulty with your balance | |  |  |  |  |  |
| Change in movement in your stronger hand | |  |  |  |  |  |
| Abnormal sleep last night | |  |  |  |  |  |
| Seizure within the last 24 hours | |  |  |  |  |  |
| Other: | |  |  |  |  |  |

Add comments in table if needed
