# Supplementary material for: Non-Invasive Brain Stimulation in Children With Unilateral Cerebral Palsy: A Protocol and Risk Mitigation Guide
Source: Front Pediatr. 2018 Mar 16;6:56. doi: 10.3389/fped.2018.00056 (PMC5864860; doi:10.3389/fped.2018.00056)
Supplement: Appendix A — Seizure Management. [file Data_Sheet_1.ZIP › Appendix_G.DOCX]

Supplementary Material

**Non-Invasive Brain Stimulation in Children with Unilateral Cerebral Palsy:**

A Protocol and Risk Mitigation Guide

Gillick BT^1*^, Gordon AM^2^, Feyma T^3^, Krach LE^4^, Carmel J^5^, Rich TL^6^, Bleyenheuft Y^7^, Friel K^5^

*** Correspondence:** Bernadette T. Gillick, Ph.D., MSPT, PT [gillick@umn.edu](mailto:gillick@umn.edu)

**Appendix G-Family Feedback Form**

(Title of XXX Study Here)

*Thank you for taking the time to complete this survey. Your feedback is important to us and will help us design future research studies. Your responses are anonymous.*

I am a Participant Caregiver

1. How satisfied were you with the XXX study? (circle a number)

1 2 3 4 5 6 7 8 9 10

Not Satisfied Completely satisfied

1. Would you do the XXX study again? Yes No

Why?

1. Would you recommend the study to others? Yes No

Why?

1. What were some of the things you liked about this study?
2. What were some of the things you disliked about this study?
3. What are some things you would change about this study?
4. How did you hear about us?
